# Solubilization and thermodynamic properties of simvastatin in various micellar solutions of different non-ionic surfactants: Computational modeling and solubilization capacity

**DOI:** 10.1371/journal.pone.0249485

**Published:** 2021-04-08

**Authors:** Faiyaz Shakeel, Sultan Alshehri, Mohamed A. Ibrahim, Mohammad Altamimi, Nazrul Haq, Ehab M. Elzayat, Gamal A. Shazly

**Affiliations:** 1 Department of Pharmaceutics, College of Pharmacy, King Saud University, Riyadh, Saudi Arabia; 2 Department of Pharmaceutics and Industrial Pharmacy, Faculty of Pharmacy, Al-Azhar University, Assiut, Egypt; 3 Department of Industrial Pharmacy, Faculty of Pharmacy, Al-Azhar University, Assiut, Egypt; University of Alcalá, SPAIN

## Abstract

The aim of this work was to solubilize simvastatin (SIM) using different micellar solutions of various non-ionic surfactants such as Tween-80 (T80), Tween-20 (T20), Myrj-52 (M52), Myrj-59 (M59), Brij-35 (B35) and Brij-58 (B58). The solubility of SIM in water (H_2_O) and different micellar concentrations of T80, T20, M52, M59, B35 and B58 was determined at temperatures *T* = 300.2 K to 320.2 K under atmospheric pressure *p* = 0.1 MPa using saturation shake flask method. The experimental solubility data of SIM was regressed using van’t Hoff and Apelblat models. The solubility of SIM (mole fraction) was recorded highest in M59 (1.54 x 10^−2^) followed by M52 (6.56 x 10^−3^), B58 (5.52 x 10^−3^), B35 (3.97 x 10^−3^), T80 (1.68 x 10^−3^), T20 (1.16 x 10^−3^) [the concentration of surfactants was 20 mM in H_2_O in all cases] and H_2_O (1.94 x 10^−6^) at *T* = 320.2 K. The same results were also recorded at each temperature and each micellar concentration of T80, T20, M52, M59, B35 and B58. “Apparent thermodynamic analysis” showed endothermic and entropy-driven dissolution/solubilization of SIM in H_2_O and various micellar solutions of T80, T20, M52, M59, B35 and B58.

## Introduction

**Fig 1:**
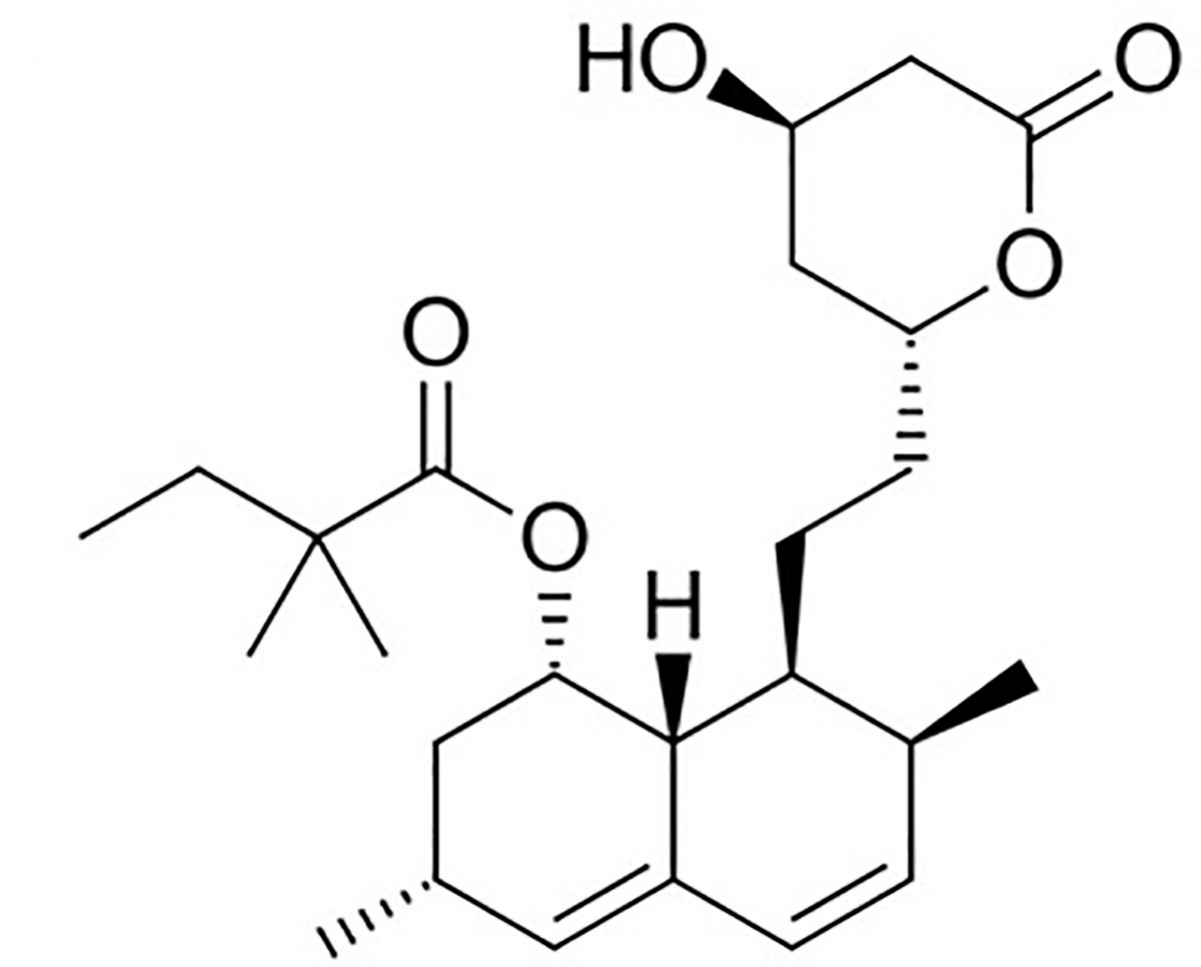
Molecular structure of simvastatin (SIM).

Simvastatin (SIM) {molecular structure: Fig 1; chemical name: [(1S,3R,7S,8S,8aR)-8-[2-[(2R,4R)-4-hydroxy-6-oxooxan-2-yl]ethyl]-3,7-dimethyl-1,2,3,7,8,8a-hexahydronaphthalen-1-yl] 2,2-dimethylbutanoate; molecular formula: C_25_H_38_O_5_; molar mass: 418.57 g mol^-1^ and CASRN: 79902-63-9) occurs as a white to off-white crystalline powder [1, 2]. It is a lipid lowering agent which belongs to statins family and powerful inhibitor of (3,5)-hydroxy-3-methylglutaryl coenzyme A (HMGr-CoA) reductase [3, 4]. Due to HMGr-CoA reductase inhibitory activity, it is used to treat and control hyper-cholesterolaemia in humans [5–7]. It shows very poor bioavailability (< 5.0%) upon oral administration which may be attributed to its poor solubility in water, low intestinal uptake and extensive first pass metabolism [2, 8, 9].

Majority of the pharmaceutical products contain one or more types of surfactants [10, 11]. Surfactants have the ability to form colloidal-sized micelles in certain liquids and hence capable in enhancing the solubility of drugs [11, 12]. Around 40% of newly synthesized active pharmaceutical ingredients lack the required aqueous solubility [13]. Therefore, surfactants have gained much interest for enhancing the solubility of poorly soluble drugs in an aqueous media [10–14]. Several solubility enhancement techniques such as cyclodextrin complexation [15, 16], solid dispersions [17–19], self-emulsifying drug delivery system [20], self-microemulsifying drug delivery system [21, 22], self-nanoemulsifying drug delivery system [8], nanoencapsulation [23, 24], supercritical fluid techniques [25] and drug-dendrimer complex [26] were investigated in solubility/dissolution enhancement of SIM. However, solid dispersion technology has been investigated most widely in solubility enhancement of SIM [2, 17–19, 27].

There is lack of temperature dependent solubility data of statins in literature. The solubilities (mole fraction) of statin drugs like lovastatin in some organic solvents such as acetone, methanol, ethanol, ethyl acetate and butyl acetate at temperature *T* = 283 K to 323 K under atmospheric pressure *p* = 0.1 MPa are reported elsewhere [28]. The solubilities of SIM (mole fraction) in various alcohols such as ethanol, 1-propanol, 1-butanol, 1-pentanol, 1-hexanol and 1-octanol at *T* = 286.15 K to 310.15 K are also available [1]. The micellar solubilization of drugs is one of the useful techniques which is being applied in solubility enhancement of weakly aqueous-soluble drug compounds [11, 29, 30]. Micellar solubilization of several poorly water-soluble drugs such as SIM, itraconazole, danazol, fenofibrate and androstane has been studied [12, 31, 32]. Temperature dependent solubilities of SIM in micellar solutions of various non-ionic surfactants such as Tween-80 (T80), Tween-20 (T20), Myrj-52 (M52), Myrj-59 (M59), Brij-35 (B35) and Brij-58 (B58) are not reported elsewhere. Therefore, the aim of this work was to determine the solubility of SIM in various molar concentrations of T80, T20, M52, M59, B35 and B58 in comparison with its solubility in water (H_2_O) at *T* = 300.2 K to 320.2 K and *p* = 0.1 MPa. The dissolution/solubilization behavior of SIM in different molar concentrations of T80, T20, M52, M59, B35 and B58 was investigated by apparent thermodynamic analysis. All studied surfactants are non-ionic surfactants which are safe for human use. They have potential for enhancing the solubility of poorly soluble drugs via micelle formation. Hence, the studied surfactants were selected for the solubilization of SIM in this work.

## Materials and methods

### Materials

SIM was obtained from Riyadh Pharmaceuticals (Riyadh, Saudi Arabia). T80 (IUPAC name: polyoxyethylene (20) sorbitan monooleate) and T20 (IUPAC name: polyoxyethylene (20) sorbitan monolaurate) were obtained from BDH Chemicals Ltd. Co. (Poole, England, UK). M52 (IUPAC name: polyoxyethylene (40) stearate), M59 (IUPAC name: polyoxyethylene (100) stearate), B35 (IUPAC name: polyoxyethylene (23) lauryl ether) and B58 (IUPAC name: polyoxyethylene (20) cetyl ether) were obtained from Sigma Aldrich (St. Louis, MO, USA). Chromatography grade acetonitrile (IUPAC name: cyanomethane) and formic acid (IUPAC name: methanoic acid) were obtained from E-Merck (Darmstadt, Germany). H_2_O of high purity (deionized H_2_O) was collected from Milli-Q Water Purification Unit.

### Quantification of SIM by UPLC-UV analysis

“Waters Acquity^®^ H-class Ultra-Performance Liquid Chromatography (UPLC)” apparatus connected with a “Waters diode-array-ultra-violet detector (DAD-UV) (Waters, MA, USA)” was applied for quantification of SIM at 237 nm. The quantification was carried out at reverse-phase isocratic elution mode using “Acquity^®^ UPLC BEH C_18_ column (2.1 x 50 mm, 1.7 μm)” which was acquired from “Waters (Waters Inc., Bedford, MA, USA)”. The binary mixture of 0.1% formic acid and acetonitrile (25:75, v/v) was used as mobile phase which was delivered with a flow rate of 0.3 mL min^-1^. The volume of injection was 1 μL. The quantification of SIM was performed at 237 nm. The column temperature was maintained at “*T* = 313.2 K”. The UPLC response of SIM was obtained at retention time of 1.12 min with a total run time of 1.5 min. The “Masslynx software” was utilized for data analysis.

### Calibration and regression

The measured UPLC response of SIM was plotted against its concentrations in order to obtain calibration and regression. The calibration plot of SIM was observed linear in the range of (10 to 500.0) ng g^-1^. The coefficient of determination (*R*^2^) and equation for regression line were recorded as 0.9990 and UPLC area = 225.43*concentration—502.98. The proposed UPLC-UV method was validated in terms of “linearity, accuracy, precision, robustness, sensitivity, reproducibility and specificity”. The results of validation parameters were obtained within the recommended limits of International Council for Harmonization guidelines [33].

### Solid state characterization of pure and SIM equilibrated with water

The solid phases of SIM in pure and equilibrated samples (equilibrated with water) were characterized by differential scanning calorimetry (DSC) and powder X-ray diffraction (PXRD) studies. The pure SIM was original SIM powder which was used before solubility studies. The equilibrated SIM was recovered from water after solubility studies. The equilibrated SIM was recovered by slow evaporation of water and stored at an ambient temperature till further use. The characterization of solid phases was performed for the investigation of physical form and probable transformation of SIM into polymorphs/solvates/hydrates after equilibrium. DSC thermogram of SIM in pure and equilibrated forms was obtained using “DSC-8000 Instrument (Perkin Elmer, MA, USA)”. The whole DSC assembly was connected with chiller and autosampler. Before DSC experiments, the calibration of instrument was performed using pure indium. Accurately weighed 5.40 mg of pure SIM and 5.20 mg of equilibrated SIM were taken and transferred into an aluminium pan which was sealed hermetically. DSC spectra for SIM in both samples was recorded in the temperature range of *T* = 303.2 K to 573.2 K with heating rate of 10.0 K min^-1^. The flow for nitrogen for this analysis was set at 20 mL min^-1^.

PXRD spectra of SIM in both samples were obtained with the help of “Ultima IV Diffractometer (Rigaku Inc. Tokyo, Japan)” in the 2θ range of 3−60° at a scan speed of 0.5° min^-1^. The tube anode utilized for PXRD measurements was “Cu with Ka = 0.1540562 nm mono chromatized with a graphite crystal (Rigaku Inc., Tokyo, Japan)”. PXRD spectra of SIM in both samples were recorded at tube voltage and tube current of 40 kV and 40 mA, respectively.

### Measurement of SIM solubility in H_2_O and various micellar solutions of different non-ionic surfactants

The solubilities of SIM (mole fraction) in H_2_O and different micellar solutions of T80, T20, M52, M59, B35 and B58 were measured using a saturation shake flask technique propose by Higuchi and Connors [34]. The solubility of SIM was measured at *T* = 300.2 K to 320.2 K under atmospheric pressure. The excess quantity of pure SIM was added into known quantities of H_2_O and various micellar solutions (1, 5, 10 and 20 mM) of T80, T20, M52, M59, B35 and B58. Each experiment was performed in triplicates manner. Each drug-surfactant/drug-H_2_O mixture was vortexed using a Vortex mixer (Thermo Fisher Scientific, Waltham, MA, USA) for about 5 min. The samples were then kept in the WiseBath^®^ WSB Shaking Water Bath (Model WSB-18/30/-45, Daihan Scientific Co. Ltd., Seoul, Korea). The speed of shaker was maintained at 100 rpm and temperature was varied from 300.2 K to 320.2 K. The equilibrium time was optimized as 72 h by preliminary investigations. After 72 h, each drug-surfactant/drug-H_2_O mixture was taken out from the WSB shaking Water bath. The samples were centrifuged using a Remi Centrifuge (Remi Sales & Eng. Ltd., Mumbai, India) at 5000 rpm for about 20 min at ambient temperature i.e. *T* = 298.2 K. The supernatants were withdrawn, filtered using Whatman filter paper (Sigma Aldrich, St. Louis, MO, USA), diluted (wherever applicable) and subjected for the quantification of SIM by UPLC-UV technique at 237 nm. The experimental mole fraction solubility (*x*_e_) values of SIM were obtained using Eq (1) [35, 36]:

$$x_{e}=\frac{m_{1}/M_{1}}{m_{1}/M_{1}+m_{2}/M_{2}}$$ (1)

In which, *m*_1_ and *m*_2_ represent the amounts of SIM (g) and H_2_O/surfactant (g), respectively. *M*_1_ and *M*_2_ represent the molecular weights of SIM (g mol^-1^) and H_2_O/surfactant (g mol^-1^), respectively.

## Results and discussion

### Solid state characterization of pure and equilibrated SIM

**Fig 2:**
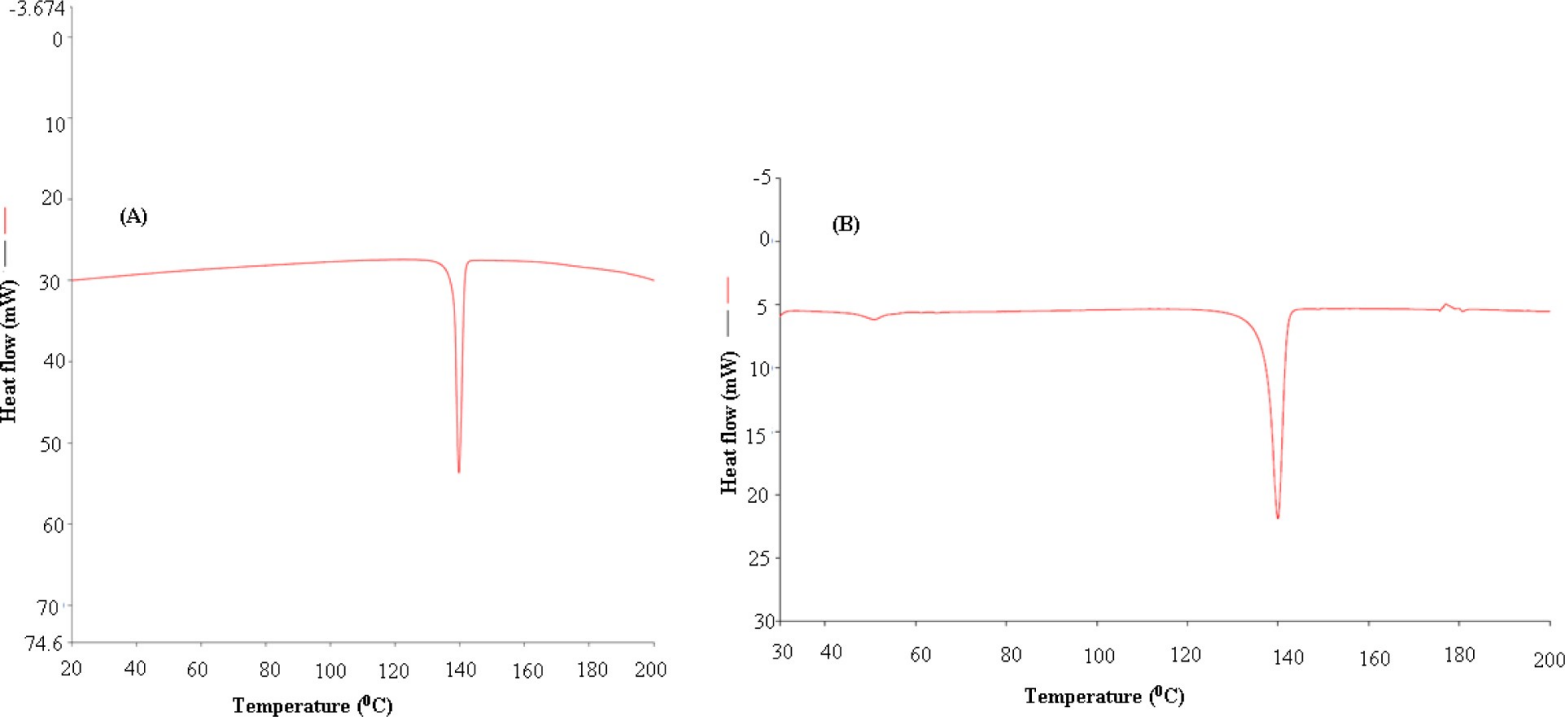
Differential scanning calorimetry (DSC) spectra of (A) pure SIM and (B) equilibrated SIM recovered from water after slow evaporation.

The solid phases of SIM in both samples were characterized for the investigation their physical form and possible transformation of SIM into polymorphs/solvates/hydrates after equilibrium. DSC thermograms of SIM in pure and equilibrated samples are shown in Fig 2A and 2B, respectively. DSC thermogram of SIM in pure form presented a crystalline endothermic peak at melting/fusion temperature (*T*_fus_) of 412.95 K. The values of fusion enthalpy (Δ*H*_fus_) and fusion entropy (Δ*S*_fus_) for pure SIM were obtained as 28.38 kJ mol^-1^ and 68.72 J mol^-1^ K^-1^, respectively (Fig 2A). The equilibrated SIM was recovered from slow evaporation of water. DSC thermogram of SIM in equilibrated form (the SIM equilibrated with water) also presented a crystalline endothermic peak at *T*_fus_ of 413.18 K. The values of Δ*H*_fus_ and Δ*S*_fus_ for equilibrated SIM were obtained as 28.58 kJ mol^-1^ and 69.19 J mol^-1^ K^-1^, respectively (Fig 2B). The DSC spectra and various thermal parameters such as *T*_fus_, Δ*H*_fus_ and Δ*S*_fus_ of pure SIM very closed with those of equilibrated SIM. The results of DSC analysis indicated crystalline nature of SIM in both samples. Although, the peak intensities of pure and equilibrated SIM were slightly different, but their thermal parameters were almost closed to each other. The difference in peak intensity might be due to the fact that different amounts of pure and equilibrated SIM were taken for DSC analysis. Similar DSC spectra for pure and equilibrated SIM suggested no transformation of SIM into amorphous/polymorphic/solvate form after equilibrium. The *T*_fus_ and Δ*H*_fus_ values of pure SIM have been reported as 410.92 K and 24.46 kJ mol^-1^, respectively [1]. The *T*_fus_ and Δ*H*_fus_ values of pure SIM were obtained as 412.95 K and 28.38 kJ mol^-1^, respectively in the present study. These thermal parameters of present work were found to be closed with literature values [1].

**Fig 3:**
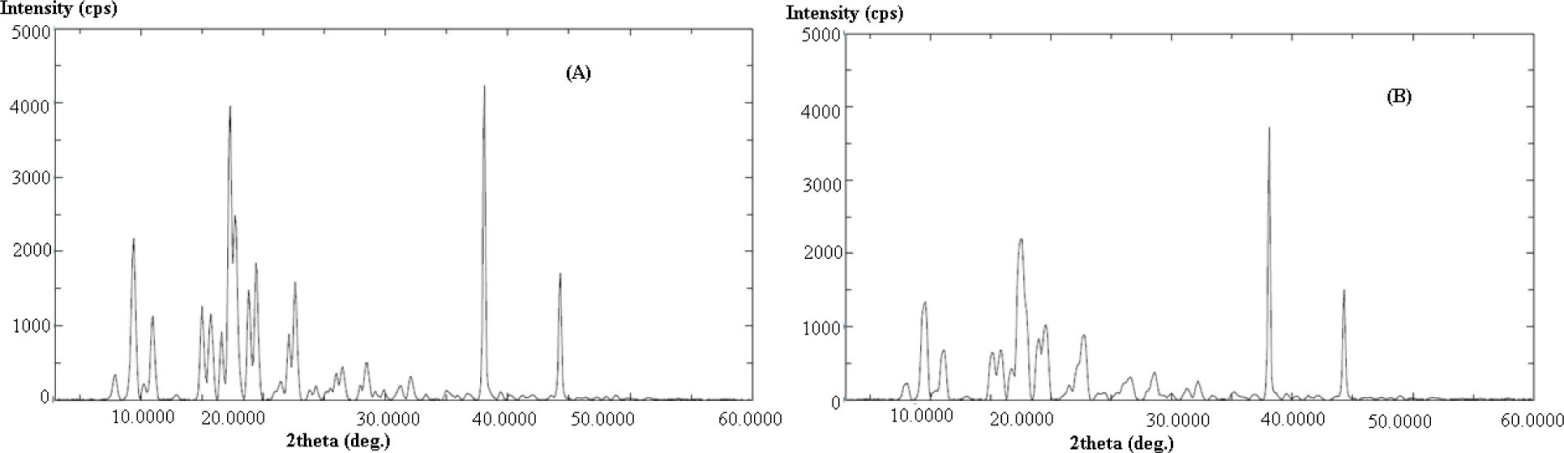
Powder X-ray diffraction (PXRD) spectra of (A) pure SIM and (B) equilibrated SIM recovered from water after slow evaporation.

The PXRD spectra of pure and equilibrated SIM are shown in Fig 3A and 3B, respectively. PXRD spectra of SIM in pure sample presented different crystalline peaks at various 2 θ values, also suggesting crystalline nature of SIM (Fig 3A). PXRD spectra of SIM in equilibrated form also presented different crystalline peaks at similar 2 θ values (Fig 3B). Similar PXRD spectra of pure and equilibrated SIM again suggested crystalline nature of SIM in both samples and no transformation of SIM into amorphous/polymorphic/solvate form after equilibrium. Based on DSC and PXRD results, we can say that the crystal form of SIM was similar in water and most probably on studied surfactants as no transformation of SIM was recorded after equilibrium.

### Experimental solubilities of SIM in H_2_O and various micellar solutions of different non-ionic surfactants

The experimental solubility (*x*_e_) values of SIM in H_2_O and various micellar solutions (1, 5, 10 and 20 mM) of T80, T20, M52, M59, B35 and B58 at three different temperatures *T* = 300.2 K, 310.2 K and 320.2 K and *p* = 0.1 MPa are presented in Table 1. Saturated solubility of SIM in H_2_O at ambient temperature i.e. *T* = 298.2 K has been reported elsewhere [19, 27]. Micellar solubilization of SIM in polyglycerol diisostearate ethoxylates surfactants has also been reported [12]. However, temperature-dependent solubilities of SIM in H_2_O and various micellar solutions of T80, T20, M52, M59, B35 and B58 are not reported so far. Murtaza reported the saturated solubility of SIM in H_2_O at *T* = 298.2 K as 30.00 μg mL^-1^ (converted to 7.57 x 10^−7^ in mole fraction) [27]. However, Craye et al. reported the saturated solubility of SIM in H_2_O at *T* = 298.2 K as 1.74 μg mL^-1^ (converted to 7.49 x 10^−8^ in mole fraction) [19]. The mole fraction solubility of SIM in H_2_O at *T* = 298.2 K was not determined directly in the present work. The mole fraction solubility of SIM in H_2_O at *T* = 298.2 K was determined from extrapolation of curve plotted between ln *x*_e_ and 1/*T* and obtained as 7.08 x 10^−7^ in our work. Solubility of SIM in H_2_O recorded in this study was much closed with that reported by Murtaza [27]. However, it was much deviated from solubility of SIM reported by Craye et al. [19].

**Table 1:** Mole fraction solubility (*x*_e_) values of simvastatin (SIM) in water (H_2_O) and various micellar solutions of different non-ionic surfactants at *T* = 300.2 K to 320.2 K and *p* = 0.1 MPa^a^.

Samples	*x*_e_		
	*T* = 300.2 K	*T* = 310.2 K	*T* = 320.2 K
H_2_O	7.57 x 10^−7^	1.29 x 10^−6^	1.94 x 10^−6^
1 mM T80	9.37 x 10^−5^	1.29 x 10^−4^	1.77 x 10^−4^
5 mM T80	5.00 x 10^−4^	6.36 x 10^−4^	8.27 x 10^−4^
10 mM T80	7.34 x 10^−4^	9.32 x 10^−4^	1.18 x 10^−3^
20 mM T80	1.08 x 10^−3^	1.35 x 10^−3^	1.68 x 10^−3^
1 mM T20	4.69 x 10^−5^	6.42 x 10^−5^	8.80 x 10^−5^
5 mM T20	1.94 x 10^−4^	2.52 x 10^−4^	3.31 x 10^−4^
10 mM T20	4.84 x 10^−4^	6.45 x 10^−4^	8.06 x 10^−4^
20 mM T20	7.18 x 10^−4^	9.13 x 10^−4^	1.16 x 10^−3^
1 mM M52	2.75 x 10^−4^	3.72 x 10^−4^	4.78 x 10^−4^
5 mM M52	1.54 x 10^−3^	1.90 x 10^−3^	2.33 x 10^−3^
10 mM M52	3.60 x 10^−3^	4.29 x 10^−3^	5.20 x 10^−3^
20 mM M52	4.67 x 10^−3^	5.62 x 10^−3^	6.56 x 10^−3^
1 mM M59	4.92 x 10^−4^	6.61 x 10^−4^	9.17 x 10^−4^
5 mM M59	2.28 x 10^−3^	2.84 x 10^−3^	3.54 x 10^−3^
10 mM M59	6.01 x 10^−3^	7.03 x 10^−3^	8.44 x 10^−3^
20 mM M59	1.15 x 10^−2^	1.33 x 10^−2^	1.54 x 10^−2^
1 mM B35	1.35 x 10^−4^	1.87 x 10^−4^	2.53 x 10^−4^
5 mM B35	3.43 x 10^−4^	5.48 x 10^−4^	8.15 x 10^−4^
10 mM B35	1.13 x 10^−3^	1.39 x 10^−3^	1.76 x 10^−3^
20 mM B35	2.71 x 10^−3^	3.25 x 10^−3^	3.97 x 10^−3^
1 mM B58	9.11 x 10^−5^	1.37 x 10^−4^	1.94 x 10^−4^
5 mM B58	2.55 x 10^−4^	3.36 x 10^−4^	4.55 x 10^−4^
10 mM B58	4.34 x 10^−4^	5.57 x 10^−4^	7.55 x 10^−4^
20 mM B58	3.74 x 10^−3^	4.47 x 10^−3^	5.52 x 10^−3^
*x*^idl^	7.16 x 10^−2^	9.39 x 10^−2^	1.22 x 10^−1^

^a^The relative uncertainties *u*_r_ are *u*_r_(*T*) = 0.016, *u*_r_(*p*) = 0.003 and *u*_r_(*x*_e_) = 0.014.

**Fig 4:**
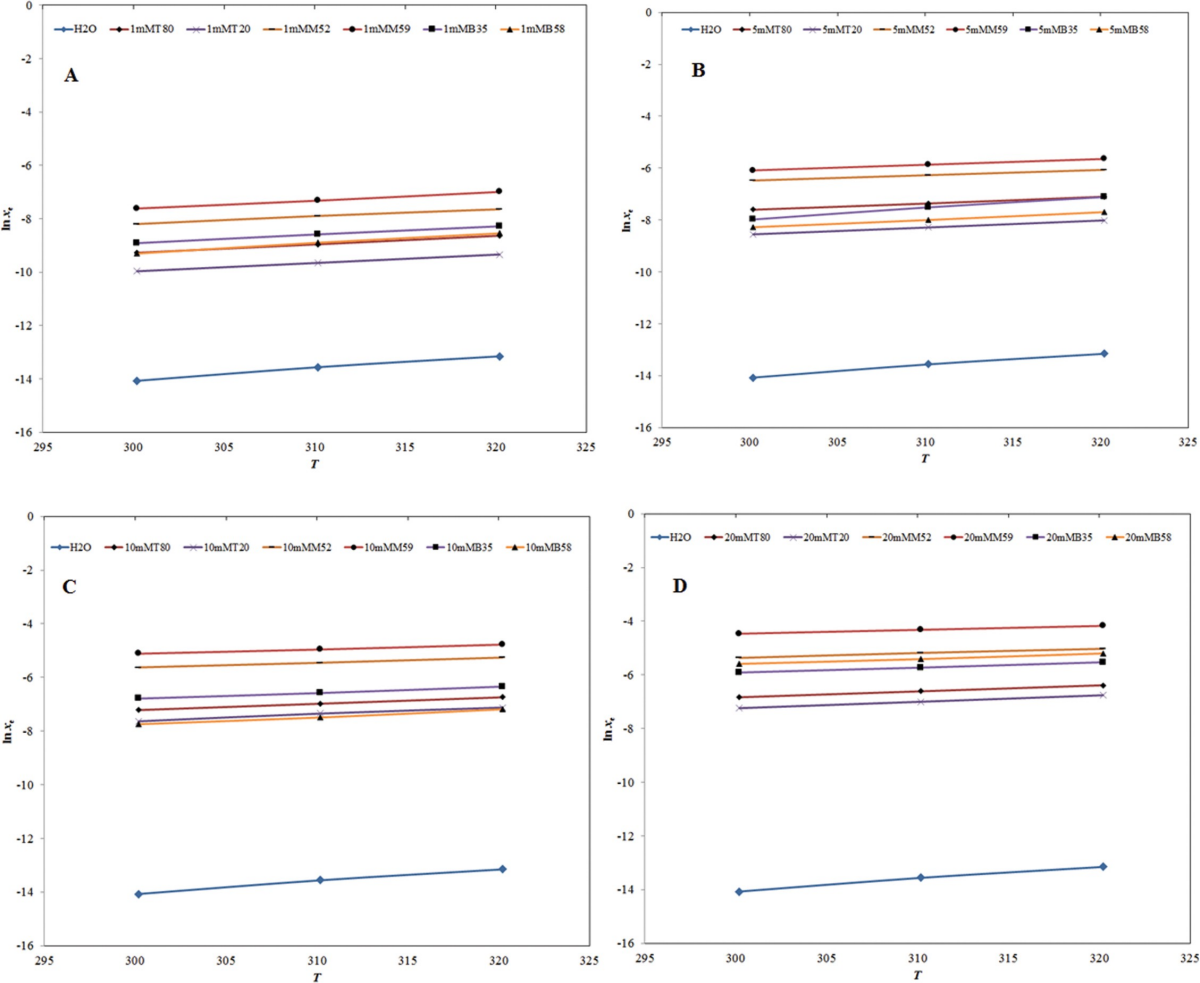
Influence of temperature on logarithmic solubility (ln *x*_e_) values of SIM in (A) H_2_O and 1 mM molar solution of various non-ionic surfactants and (B) H_2_O and 5 mM molar solution of various non-ionic surfactants. Influence of temperature on ln *x*_e_ values of SIM in (C) H_2_O and 10 mM molar solution of various non-ionic surfactants and (D) H_2_O and 20 mM molar solution of various non-ionic surfactants.

The influence of temperature on logarithmic solubilities of SIM is presented in Fig 4. It was observed from experimental data that the logarithmic solubility values of SIM were increasing linearly with increase in temperature in H_2_O and four different micellar solutions of T80, T20, M52, M59, B35 and B58 (Fig 4). The results of influence of temperature on solubility of SIM were accordance with those reported for several weakly water soluble drugs [35–39].

**Fig 5:**
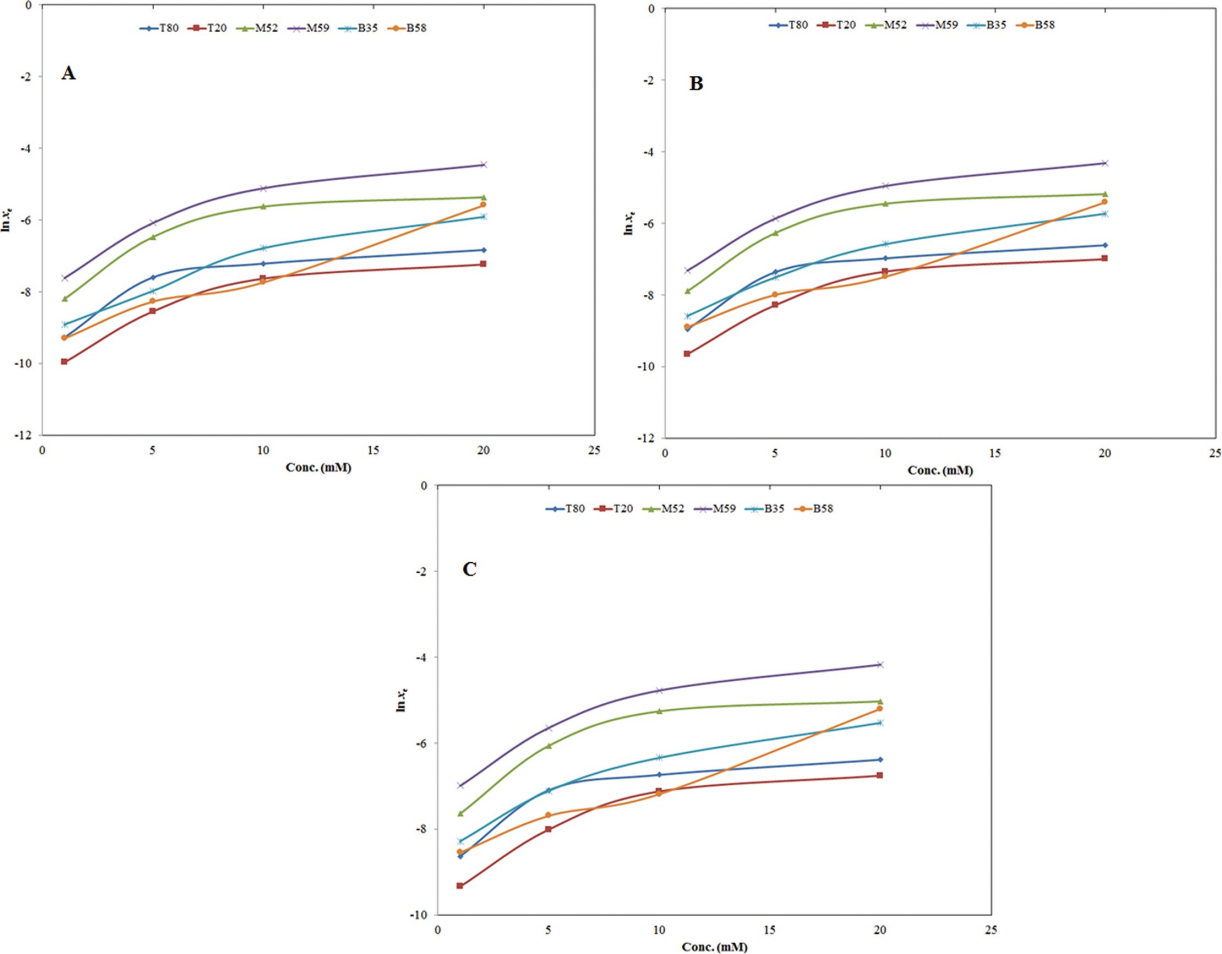
Influence of molar concentrations of different non-ionic surfactants on ln *x*_e_ values of SIM at (A) *T* = 300.2 K, (B) *T* = 310.2 K and (C) *T* = 320.2 K.

The influence of molar concentrations of various non-ionic surfactants on logarithmic solubilities of SIM at three different temperatures is presented in Fig 5. It was found that the logarithmic solubility values of SIM were increasing non-linearly with increase in the molar concentrations of T80, T20, M52, M59, B35 and B58 at each temperature studied. The *x*_e_ values of SIM recorded highest in M59 (1.54 x 10^−2^) followed by M52 (6.56 x 10^−3^), B58 (5.52 x 10^−3^), B35 (3.97 x 10^−3^), T80 (1.68 x 10^−3^), T20 (1.16 x 10^−3^) [the concentration of surfactants was 20 mM in H_2_O in all cases] and H_2_O (1.94 x 10^−6^) at *T* = 320.2 K. The same results were also obtained at each temperature and four different micellar solutions of T80, T20, M52, M59, B35 and B58. The *x*_e_ values of SIM were much higher in M59 in comparison with H_2_O. The maximum *x*_e_ values of SIM in M59 might be possible due to similar polarity of SIM and M59. Due to the highest solubility of SIM in 20 mM M59, it can be used as a solubilizer in liquid formulation design of SIM.

### Solubility parameter for SIM, H_2_O and different surfactants

In this work, Hansen solubility parameter (*δ*) for SIM, H_2_O, T80, T20, M52, M59, B35 and B58 was obtained using Eq (2) [40–42]:

$$\delta^{2}=\delta_{d}^{2}+\delta_{p}^{2}+\delta_{h}^{2}$$ (2)

In which, the symbol *δ* is the total Hansen solubility parameter for solute/solvent. However, the symbols *δ*_d_, *δ*_p_ and *δ*_h_ represent dispersion, polar and hydrogen-bonded Hansen solubility parameters, respectively. The *δ*, *δ*_d_, *δ*_p_ and *δ*_h_ values were obtained by putting “simplified molecular-input line-entry system (SMILES)” of each component using “HSPiP software (version 4.1.07)” The SMILES of each compound is easily available in the compound database. The calculated values of *δ*, *δ*_d_, *δ*_p_ and *δ*_h_ are presented in Table 2. From “HSPiP software”, the value of *δ* for SIM was obtained as 18.70 MPa^1/2^ which suggesting that SIM had lower polarity. The *δ* value for three different non-ionic surfactants i.e. M52, M59 and B58 was recorded as 18.70 MPa^1/2^. However, the value of *δ* for T80, T20, B35 and H_2_O was obtained as 21.30, 22.10, 18.90 and 47.80 MPa^1/2^, respectively. The *x*_e_ values of SIM were obtained higher in M59, M52 and B35 which was possible due to same *δ* values for SIM, M59, M52 and B58 (Table 2). However, the *x*_e_ value of SIM was recorded lowest in H_2_O which attributed the maximum *δ* value (47.80 MPa^1/2^) of H_2_O. Overall, the results of Hansen solubility parameters suggested good agreement of experimental solubility data of SIM with their polarities/solubility parameters.

**Table 2:** Hansen solubility parameters for SIM, H_2_O and different non-ionic surfactants at *T* = 298.2 K calculated using HSPiP software.

Components	Hansen solubility parameters			
	*δ*_d_/MPa^1/2^	*δ*_p_/MPa^1/2^	*δ*_h_/MPa^1/2^	*δ*/MPa^1/2^
SIM	46.60	6.60	5.70	18.70
T80	14.80	8.60	12.70	21.30
T20	14.90	9.40	13.30	22.10
M52	16.10	3.90	7.90	18.40
M59	16.10	3.90	7.90	18.40
B35	9.00	9.70	13.50	18.90
B58	10.10	8.80	12.60	18.40
H_2_O	15.50	16.00	42.30	47.80

### Determination of drug solubilization efficiency

The drug solubilization efficiency for different micellar solutions of various non-ionic surfactants was determined as the molar solubilization capacity (*S*_c_) using Eq (3) [31, 32]:

$$S_{c}=\frac{S_{t}-S_{W}}{C_{S}-CMC}\times 1000$$ (3)

In which, *S*_t_ is the measured SIM solubility in the presence of surfactants, *S*_w_ is the intrinsic water solubility of SIM, *C*_s_ is the molar surfactant concentration and CMC is the critical micelle concentration of surfactant. The values of solubilization capacity for SIM in different micellar solutions of various non-ionic surfactants were determined at “*T* = 300.2 K” and results are presented in Table 3. The solubilization capacity for SIM was found to be lower in all micellar solutions of T80, T20, B35 and B58 compared to various micellar solutions of M52 and M59. The best solubilization capacity (*x* = 174.0) was found in 10 mM micellar solution of M52.

**Table 3:** SIM solubilization capacity in various micellar solutions of different non-ionic surfactants at *T* = 300.2 K.

Surfactant	Solubilization capacity (mM M^-1^)
1 mM T80	29.40
5 mM T80	68.10
10 mM T80	51.90
20 mM T80	39.10
1 mM T20	51.30
5 mM T20	23.30
10 mM T20	35.30
20 mM T20	27.20
1 mM M52	102.0
5 mM M52	145.0
10 mM M52	174.0
20 mM M52	113.0
1 mM M59	62.60
5 mM M59	89.00
10 mM M59	125.0
20 mM M59	122.0
1 mM B35	72.90
5 mM B35	49.30
10 mM B35	90.60
20 mM B35	112.0
1 mM B58	41.50
5 mM B58	37.40
10 mM B58	34.70
20 mM B58	166.0

### Theoretical/ideal solubilities

Theoretical/ideal solubility of solute/SIM (*x*^idl^) was obtained using Eq (4) [43, 44]:

$$lnx^{idl}=\frac{-\Delta H_{fus}(T_{fus}-T)}{RT_{fus}T}+(\frac{{\Delta C}_{p}}{R})[\frac{T_{fus}-T}{T}+\ln\left(\frac{T}{T_{fus}}\right)]$$ (4)

In which, *R* represents the universal gas constant and Δ*C*_p_ represents the differential molar heat capacity of solute/SIM [43–45]. Other symbols in Eq (4) were defined previously in the article.

The values of *T*_fus_, Δ*H*_fus_ and Δ*C*_p_ for solute/SIM were obtained as 412.95 K, 28.38 kJ mol^-1^ and 68.72 J mol^-1^ K^-1^, respectively from DSC/thermal analysis of SIM. The *x*^idl^ values for solute/SIM were obtained using Eq (4) and these values at three different temperatures are presented in Table 1. Theoretical/ideal solubilities of SIM were compared with experimental solubilities at each temperature. It was noticed that theoretical/ideal solubility of SIM was significantly higher than SIM solubility in H_2_O and various micellar solutions (1, 5, 10 and 20 mM) of T80, T20, M52, M59, B35 and B58 at each temperature investigated. Theoretical/ideal solubility of SIM was also recorded as increasing significantly with increase in temperature, suggesting the dissolution behavior of SIM was endothermic process [1].

### Model solubilities and curve fitting

The experimental solubilities of SIM were modelled/curve fitted with the help of van’t Hoff and Apelblat models [38, 46, 47]. Apelblat model solubility (*x*^Apl^) of SIM in H_2_O and various micellar solutions (1, 5, 10 and 20 mM) of T80, T20, M52, M59, B35 and B58 was calculated using of Eq (5) [46, 47]:

$$lnx^{Apl}=A+\frac{B}{T}+C\ln\left(T\right)$$ (5)

In which, *A*, *B* and *C* represent the coefficients/parameters of Apelblat model which were obtained by applying “nonlinear multivariate regression analysis” of experimental solubilities of SIM listed in Table 1 [48]. The *x*_e_ of SIM were modelled/curve fitted with Apelblat solubilities of SIM using root mean square deviations (*RMSD*) and *R*^2^. *RMSD* values between experimental and Apelblat solubilities of SIM were obtained using Eq (6) [35]:

$$RMSD={\left[\frac{1}{N}\sum_{i=1}^{N}{\left(\frac{x^{Apl}-x_{e}}{x_{e}}\right)}^{2}\right]}^{\frac{1}{2}}$$ (6)

**Fig 6:**
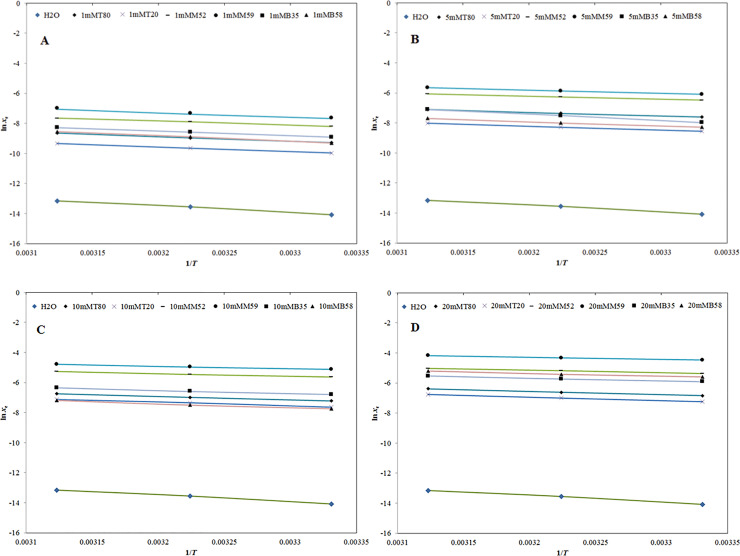
Correlation of ln *x*_e_ values of SIM with “Apelblat model” in (A) H_2_O and 1 mM molar solution of various non-ionic surfactants and (B) H_2_O and 5 mM molar solution of various non-ionic surfactants as a function of 1/*T*; symbols represent the experimental solubilities of SIM and solid lines represent the solubilities of SIM calculated by “Apelblat model”. Correlation of ln *x*_e_ values of SIM with “Apelblat model” in (C) H_2_O and 10 mM molar solution of various non-ionic surfactants and (D) H_2_O and 20 mM molar solution of various non-ionic surfactants as a function of 1/*T*; symbols represent the experimental solubilities of SIM and solid lines represent the solubilities of SIM calculated by “Apelblat model”.

In which, *N* represents the number of experimental data points used in the study. The graphical correlation/curve fitting between logarithmic experimental solubilities (ln *x*_e_) and logarithmic Apelblat solubilities (ln *x*^Apl^) of SIM in H_2_O and 1 mM and 5 mM micellar solution of T80, T20, M52, M59, B35 and B58 against reciprocal of absolute temperature (1/*T*) is presented in Fig 6A and 6B, respectively.

However, the curve fitting between ln *x*_e_ and ln *x*^Apl^ of SIM in H_2_O and 10 mM and 20 mM micellar solution of T80, T20, M52, M59, B35 and B58 against 1/*T* is presented in Fig 6C and 6D, respectively. The results showed in Fig 6A–6D suggested good correlation/curve fitting between ln *x*_e_ and ln *x*^Apl^ values of SIM in H_2_O and different micellar solutions of T80, T20, M52, M59, B35 and B58. The resulting data of this correlation/fitting are listed in Table 4. *RMSD* values for SIM in H_2_O and various micellar solutions of T80, T20, M52, M59, B35 and B58 were obtained as (0.16 to 5.84) %. An average *RMSD* for this correlation was found to be 0.60%. The *R*^2^ values for SIM in H_2_O and various micellar solutions of T80, T20, M52, M59, B35 and B58 were obtained in the range of 0.9957 to 0.9999. The results presented in Table 4 in terms of *RMSD* and *R*^2^ suggested good correlation of experimental data of SIM with Apelblat model.

**Table 4:** The parameters of Apelblat model (*A*, *B* and *C*) along with determination coefficient (*R*^2^) and root mean square deviation (% *RMSD*) for SIM in H_2_O and various micellar solutions of different non-ionic surfactants.

Samples	*A*	*B*	*C*	*R*^2^	*RMSD* (%)	Overall *RMSD* (%)
H_2_O	647.42	-34175.10	-96.00	0.9968	0.64	
1 mM T80	-181.54	5330.48	27.08	0.9998	0.37	
5 mM T80	-311.63	11940.19	46.32	0.9987	0.43	
10 mM T80	-103.18	2473.20	15.37	0.9999	0.24	
20 mM T80	-113.47	3086.68	16.89	0.9999	0.33	
1 mM T20	-155.24	4127.85	23.05	0.9999	0.44	
5 mM T20	-176.33	5533.73	26.18	0.9998	0.23	
10 mM T20	408.99	-21253.40	-60.62	0.9960	0.37	
20 mM T20	-127.46	3570.18	18.99	0.9999	0.21	
1 mM M52	309.92	-16896.90	-45.90	0.9978	0.28	
5 mM M52	-85.63	1970.57	12.72	0.9999	0.22	0.60
10 mM M52	-248.25	9672.29	36.88	0.9984	0.26	
20 mM M52	171.06	-9500.15	-25.38	0.9982	0.16	
1 mM M59	-410.22	15997.45	61.23	0.9985	5.84	
5 mM M59	-108.27	2914.14	16.21	0.9999	0.46	
10 mM M59	-299.01	12145.24	44.42	0.9970	0.57	
20 mM M59	-73.76	2004.46	10.97	0.9999	0.27	
1 mM B35	74.36	-6399.34	-10.86	0.9998	0.22	
5 mM B35	391.42	-21901.50	-57.22	0.9985	0.53	
10 mM B35	-401.65	16360.83	59.66	0.9969	0.81	
20 mM B35	-243.08	9359.92	36.11	0.9986	0.19	
1 mM B58	307.96	-17678.60	-48.29	0.9987	0.31	
5 mM B58	-341.94	12988.89	50.90	0.9988	0.65	
10 mM B58	-575.75	23887.41	85.62	0.9957	0.40	
20 mM B58	-364.22	14921.47	54.15	0.9966	0.60	

The van’t Hoff model solubility (*x*^van’t^) of SIM in H_2_O and various micellar solutions (1, 5, 10 and 20 mM) of T80, T20, M52, M59, B35 and B58 was obtained using Eq (7) [38]:

$$lnx^{van\prime t}=a+\frac{b}{T}$$ (7)

In which, *a* and *b* represent the coefficients/parameters of van’t Hoff model which were obtained by least square method.

The experimental solubilities of SIM were modelled/curve fitted with van’t Hoff solubilities of SIM using *RMSD* and *R*^2^. The curve fitting between logarithmic experimental solubilities and logarithmic van’t Hoff solubilities of SIM in H_2_O and 1 mM and 5 mM micellar solution of T80, T20, M52, M59, B35 and B58 against 1/*T* is shown in S1 and S2 Figs, respectively. However, the curve fitting between logarithmic experimental solubilities and logarithmic van’t Hoff solubilities of SIM in H_2_O and 10 mM and 20 mM micellar solution of T80, T20, M52, M59, B35 and B58 against 1/*T* is presented in S3 and S4 Figs, respectively. The data presented in S1–S4 Figs also showed good correlation/curve fitting between experimental and model solubilities of SIM in H_2_O and different micellar solutions of T80, T20, M52, M59, B35 and B58. The resulting data of this correlation are presented in Table 5. The *RMSD* values for SIM in H_2_O and various micellar solutions of T80, T20, M52, M59, B35 and B58 were obtained as (0.23 to 1.74) %. An average *RMSD* for this correlation was predicted as 0.78%. The *R*^2^ values for SIM in H_2_O and various micellar solutions of T80, T20, M52, M59, B35 and B58 were recorded as 0.9944 to 1.0000. The results presented in Table 5 in terms of *RMSD* and *R*^2^ again suggested good correlation of experimental data of SIM with van’t Hoff model.

**Table 5:** The parameters of van’t Hoff model (*a* and *b*) along with *R*^2^ and % *RMSD* for SIM in H_2_O and various micellar solutions of different non-ionic surfactants.

Samples	*a*	*B*	*R*^2^	*RMSD* (%)	Overall *RMSD* (%)
H_2_O	0.62	-4407.80	0.9977	1.74	
1 mM T80	0.93	-3066.30	0.9995	0.57	
5 mM T80	0.45	-2421.40	0.9979	0.90	
10 mM T80	0.42	-2294.20	0.9997	0.31	
20 mM T80	0.32	-2149.50	0.9996	0.33	
1 mM T20	0.08	-3019.90	0.9996	0.51	
5 mM T20	0.04	-2582.60	0.9994	0.57	
10 mM T20	0.55	-2456.10	0.9971	1.12	
20 mM T20	0.47	-2316.80	0.9996	0.36	
1 mM M52	0.68	-2664.80	0.9986	0.84	
5 mM M52	0.10	-1974.60	0.9997	0.37	0.78
10 mM M52	0.24	-1763.00	0.9975	0.68	
20 mM M52	0.06	-1630.50	0.9989	0.46	
1 mM M59	2.32	-2987.20	0.9976	1.23	
5 mM M59	0.94	-2111.70	0.9996	0.53	
10 mM M59	0.30	-1628.80	0.9959	0.87	
20 mM M59	0.19	-1398.90	0.9996	0.25	
1 mM B35	1.18	-3030.50	1.0000	0.23	
5 mM B35	5.87	-4157.40	0.9991	1.14	
10 mM B35	0.32	-2137.80	0.9957	1.02	
20 mM B35	0.19	-1835.30	0.9978	0.72	
1 mM B58	2.80	-3634.10	0.9993	0.84	
5 mM B58	1.02	-2793.60	0.9981	1.30	
10 mM B58	1.10	-2658.80	0.9944	1.71	
20 mM B58	0.62	-1868.60	0.9954	1.07	

### Apparent thermodynamics

Apparent thermodynamics is helpful in evaluation of various thermodynamic parameters, which could ultimately determine the dissolution behavior in case of real solutions and solubilization in case of non-ideal solutions [49]. Hence, the dissolution/solubilization behavior of SIM in H_2_O and various micellar solutions of T80, T20, M52, M59, B35 and B58 were determined by applying “apparent thermodynamic analysis” on solubilities (mole fraction) of SIM. Accordingly, three different thermodynamic parameters including “apparent standard dissolution enthalpy (Δ_sol_*H*^0^), apparent standard Gibbs free energy (Δ_sol_*G*^0^) and apparent standard dissolution entropy (Δ_sol_*S*^0^)” for SIM dissolution/solubilization were determined using this analysis. The Δ_sol_*H*^0^ values for SIM dissolution/solubilization in H_2_O and various micellar solutions of T80, T20, M52, M59, B35 and B58 were determined at mean harmonic temperature (*T*_hm_) by applying van’t Hoff analysis using Eq (8) [43, 49]:

(∂lnxe∂(1T−1Thm))P=−ΔsolH0R (8)

The value of *T*_hm_ was calculated as 309.98 K using its reported formula [41]. The Δ_sol_*H*^0^ values for SIM dissolution/solubilization in H_2_O and various micellar solutions of T80, T20, M52, M59, B35 and B58 were obtained by van’t Hoff plots plotted between ln *x*_e_ values of SIM and 1T−1Thm.

The Δ_sol_*G*^0^ values for dissolution/solubilization behavior of SIM in H_2_O and various micellar solutions of T80, T20, M52, M59, B35 and B58 were also obtained at *T*_hm_ of 309.98 K by Krug et al. analysis with the help of Eq (9) [50]:

$$\Delta_{sol}G^{0}=-RT_{hm}\times intercept$$ (9)

In which, the intercept value for SIM in H_2_O and various micellar solutions of T80, T20, M52, M59, B35 and B58 was calculated from van’t Hoff plot discussed under van’t Hoff analysis.

Finally, the Δ_sol_*S*^0^ values for dissolution/solubilization behavior of SIM were obtained using the combined approaches of van’t Hoff and Krug et al. analysis with the help of Eq (10) [43, 49, 50]:

$$\Delta_{sol}S^{0}=\frac{\Delta_{sol}H^{0}-\Delta_{sol}G^{0}}{T_{hm}}$$ (10)

The calculated values of these thermodynamic parameters for dissolution/solubilization behavior of SIM in H_2_O and different micellar solutions of T80, T20, M52, M59, B35 and B58 at *T*_hm_ of 309.98 K are presented in Table 6.

**Table 6:** Apparent thermodynamic quantities (Δ_sol_*H*^0^, Δ_sol_*G*^0^ and Δ_sol_*S*^0^) along with *R*^2^ values for SIM in H_2_O and various micellar solutions of different non-ionic surfactants at *T*_hm_ of 309.98 K^a^.

Samples	Δ_sol_*H*^0^/kJ mol^-1^	Δ_sol_*G*^0^/kJ mol^-1^	Δ_sol_*S*^0^/J mol^-1^ K^-1^	*R*^2^
H_2_O	36.64	35.03	5.17	0.9977
1 mM T80	25.48	23.08	7.76	0.9995
5 mM T80	20.12	18.95	3.79	0.9979
10 mM T80	19.07	17.98	3.50	0.9997
20 mM T80	17.86	17.30	2.68	0.9996
1 mM T20	25.10	24.87	0.72	0.9996
5 mM T20	21.46	21.34	0.39	0.9994
10 mM T20	20.41	18.98	4.60	0.9971
20 mM T20	19.25	18.03	3.94	0.9996
1 mM M52	22.15	20.39	5.67	0.9986
5 mM M52	16.41	16.15	0.83	0.9997
10 mM M52	14.65	14.03	2.00	0.9975
20 mM M52	13.55	13.38	0.55	0.9989
1 mM M59	24.86	18.84	19.32	0.9976
5 mM M59	17.55	15.11	7.86	0.9996
10 mM M59	13.53	12.75	2.52	0.9959
20 mM M59	11.62	11.13	1.60	0.9996
1 mM B35	25.19	22.14	9.81	1.0000
5 mM B35	34.55	19.41	48.85	0.9991
10 mM B35	17.77	16.93	2.70	0.9957
20 mM B35	15.25	14.74	1.64	0.9978
1 mM B58	30.20	22.97	23.32	0.9993
5 mM B58	23.22	20.58	8.49	0.9981
10 mM B58	22.10	19.23	9.15	0.9943
20 mM B58	15.53	13.91	5.21	0.9954

^a^The relative uncertainties are *u*(Δ_sol_*H*^0^) = 0.30, *u*(Δ_sol_*G*^0^) = 0.26 and *u*(Δ_sol_*S*^0^) = 1.40

The Δ_sol_*H*^0^ values for SIM dissolution/solubilization in H_2_O and various micellar solutions of T80, T20, M52, M59, B35 and B58 were recorded as (11.62 to 36.64) kJ mol^-1^. The Δ_sol_*H*^0^ value for SIM dissolution was recorded highest in H_2_O (36.64 kJ mol^-1^). However, the lowest Δ_sol_*H*^0^ value (11.62 kJ mol^-1^) for SIM solubilization was obtained in 20 mM micellar concentration of M59. Overall, the low values of Δ_sol_*H*^0^ were obtained at each micellar concentration of M59 investigated. The average value of Δ_sol_*H*^0^ for SIM dissolution/solubilization was found out 20.94 kJ mol^-1^ with uncertainty of 0.30. The lowest Δ_sol_*H*^0^ value for SIM solubilization in 20 mM micellar concentration of M59 was possible due to the highest solubility (mole fraction) of SIM in 20 mM micellar concentration of M59. While, the highest Δ_sol_*H*^0^ value for SIM dissolution in H_2_O was attributed to the lowest solubility of SIM in H_2_O. The Δ_sol_*G*^0^ values for SIM dissolution/solubilization in H_2_O and various micellar solutions of T80, T20, M52, M59, B35 and B58 were recorded as (11.13 to 35.03) kJ mol^-1^. The Δ_sol_*G*^0^ value for SIM dissolution was also recorded highest in H_2_O (35.03 kJ mol^-1^). However, the lowest Δ_sol_*G*^0^ value (11.13 kJ mol^-1^) for SIM solubilization was obtained in 20 mM micellar concentration of M59. Overall, the low values of Δ_sol_*G*^0^ were also obtained at each micellar concentration of M59 investigated. The average value of Δ_sol_*G*^0^ for SIM dissolution/solubilization was found out 18.68 kJ mol^-1^ with uncertainty of 0.26. In comparison, lower values of Δ_sol_*H*^0^ and Δ_sol_*G*^0^ were obtained in 20 mM micellar concentration of M59, indicating that minimum energies are used for the solubilization of SIM in M59. The results of enthalpy and Gibbs free energy measurements were in accordance with solubility data of SIM in H_2_O and various micellar solutions of different non-ionic surfactants. The positive values of apparent standard enthalpy (Δ_sol_*H*^0^ > 0) and apparent standard Gibbs energy (Δ_sol_*G*^0^ > 0) in all samples suggested an endothermic dissolution/solubilization behavior of SIM in H_2_O and various micellar solutions of T80, T20, M52, M59, B35 and B58 [38, 51]. The positive values of Δ_sol_*H*^0^ and Δ_sol_*G*^0^ might be due to the formation of new bond energy of attraction between the drug and solvent molecules [49]. The Δ_sol_*S*^0^ values for SIM dissolution/solubilization in H_2_O and different micellar solutions of T80, T20, M52, M59, B35 and B58 were also recorded as positive values in the range of (0.39 to 48.55) J mol^-1^ K^-1^. The average Δ_sol_*S*^0^ value for SIM dissolution/solubilization was recorded as 7.28 J mol^-1^ K^-1^ with uncertainty of 1.40. The positive Δ_sol_*S*^0^ values for SIM showed an entropy-driven dissolution/solubilization behavior of SIM in H_2_O and various micellar solutions of T80, T20, M52, M59, B35 and B58 [51]. Finally, the dissolution/solubilization behavior of SIM was found to be endothermic and entropy-driven in H_2_O and various micellar solutions of T80, T20, M52, M59, B35 and B58 [36, 38, 51].

## Conclusions

The objective of this work was to solubilize SIM using different micellar solutions of various non-ionic surfactants including T80, T20, M52, M59, B35 and B58. The solubility (mole fraction) of SIM in H_2_O and various micellar solutions of T80, T20, M52, M59, B35 and B58 was determined at three different temperatures i.e. *T* = 300.2 K, 310.2 K and 320.2 K under atmospheric pressure. The results of DSC and PXRD analysis suggested crystalline nature of SIM before and after equilibrium. The solubilities (mole fraction) of SIM were regressed well with van’t Hoff and Apelblat equations. With increase in temperature, the solubility of SIM was found to be enhanced significantly in H_2_O and various micellar solutions of T80, T20, M52, M59, B35 and B58. The solubility of SIM (mole fraction) was recorded highest in M59 (20 mM) followed by M52 (20 mM), B58 (20 mM), B35 (20 mM), T80 (20 mM), T20 (20 mM) and H_2_O at *T* = 320.2 K. The same results were also recorded at each temperature and four different micellar solutions of T80, T20, M52, M59, B35 and B58. The results of “apparent thermodynamic analysis” showed an endothermic and entropy-driven dissolution/solubilization of SIM in H_2_O and various micellar solutions of T80, T20, M52, M59, B35 and B58. Overall, these results suggested that various micellar solution of non-ionic surfactants could be successfully used in solubilization of poorly water soluble drugs such as SIM.

## Supporting information

S1 Fig Correlation of ln *x*_e_ values of SIM with van’t Hoff model in H_2_O and 1 mM molar solution of various non-ionic surfactants as a function of 1/*T*; symbols represent the experimental solubilities of SIM and solid lines represent the solubilities of SIM calculated by van’t Hoff model. (DOCX)

S2 Fig Correlation of ln *x*_e_ values of SIM with van’t Hoff model in H_2_O and 5 mM molar solution of various non-ionic surfactants as a function of 1/*T*; symbols represent the experimental solubilities of SIM and solid lines represent the solubilities of SIM calculated by van’t Hoff model. (DOCX)

S3 Fig Correlation of ln *x*_e_ values of SIM with van’t Hoff model in H_2_O and 10 mM molar solution of various non-ionic surfactants as a function of 1/*T*; symbols represent the experimental solubilities of SIM and solid lines represent the solubilities of SIM calculated by van’t Hoff model. (DOCX)

S4 Fig Correlation of ln *x*_e_ values of SIM with van’t Hoff model in H_2_O and 20 mM molar solution of various non-ionic surfactants as a function of 1/*T*; symbols represent the experimental solubilities of SIM and solid lines represent the solubilities of SIM calculated by van’t Hoff model. (DOCX)

## References

[1] Aceves-Hernandez JM, Hinojosa-Torres J, Nicolas-Vazquez I, Ruvalcaba RM, Garcia RML. Solubility of simvastatin: a theoretical and experimental study. J Mol Str. 2011; 995: 41–50.

[2] de Vargas MRW, Raffin FN, Moura TFAL. Strategies used for to improve aqueous solubility of simvastatin: a systematic review. Rev Cien Farm Bas Appl. 2012; 33: 497–507.

[3] Istvan ES, Deisenhofer J. Structural mechanism for statin inhibition of HMG-CoA reductase. Science. 2001; 292: 1160–1164. 10.1126/science.1059344 11349148

[4] Istvan E. Statin inhibition of HMG-CoA reductase: a 3-dimensional view. Atheroscler Suppl. 2003; 4: 3–8. 10.1016/s1567-5688(03)00003-5 12714031

[5] Todd PA, Goa KL. Simvastatin. A review of its pharmacological and properties and therapeutic potential in hypercholesterolaemia. Drugs. 1990; 40: 583–607. 10.2165/00003495-199040040-00007 2083515

[6] Schachter M. Chemical, pharmacokinetic and pharmacodynamic properties of statins: an update. Fun Clin Pharmacol. 2005; 19: 117–125. 10.1111/j.1472-8206.2004.00299.x 15660968

[7] Edwards JE, Moore RA. Statins in hypercholesterolaemia: a dose-specific meta-analysis of lipid changes in randomized, double blind trials. BMC Family Prac. 2003; 4: E18. 10.1186/1471-2296-4-18 PMC317299 14969594

[8] Mahmoud H, Al-Suwayeh S, Elkadi S. Design and optimization of self-nanoemulsifying drug delivery systems of simvastatin aiming dissolution enhancement. Afr J Pharm Pharmacol. 2013; 7: 1482–1500.

[9] Geboers S, Stappaerts J, Tack J, Annaert P, Augustijns P. In vitro and in vivo investigation of the gastrointestinal behavior of simvastatin. Int J Pharm. 2016; 510: 296–303. 10.1016/j.ijpharm.2016.06.048 27340029

[10] Mall S, Buckton G, Rawlins D. Dissolution behavior of sulphonamides into sodium dodecyl sulfate micelles: a thermodynamic approach. J Pharm Sci. 1996; 85: 75–78. 10.1021/js950225l 8926588

[11] Rangel-Yagui CO, Pessoa A Jr, Tavares LC. Micellar solubilization of drugs. J Pharm Pharm Sci. 2005; 8: 147–163. 16124926

[12] Ding Z, Hao A, Zhang P. Surface properties and solubility of simvastatin in the micelles of polyglycerol diisostearate ethoxylates. J Disp Sci Technol. 2007; 28: 495–500.

[13] Merisko-Liversidge EM, Liversidge GG. Drug nanoparticles: formulating poorly water-soluble compounds. Toxicol Pathol. 2008; 36: 43–48. 10.1177/0192623307310946 18337220

[14] Vasconcelos T, Sarmento B, Costa P. Solid dispersions as strategy to improve oral bioavailability of poorly water soluble drugs. Drug Discov Today. 2007; 12: 1068–1075. 10.1016/j.drudis.2007.09.005 18061887

[15] Vyas A, Saraf S, Saraf S. Encapsulation of cyclodextrin complexed simvastatin in chitosan nanocarriers: a novel technique for oral delivery. J Incl Phen Macrocyc Chem. 2010; 66: 251–259.

[16] Ungaro F, Giovino C, Catanzano O, Miro A, Mele A, Quaglia F, La Rotonda MI. Use of cyclodextrins as solubilizing agents for simvastatin: Effect of hydroxypropyl-ß-cyclodextrin on lactone/hydroxyacid aqueous equilibrium. Int J Pharm. 2011; 404: 49–56, 2011. 10.1016/j.ijpharm.2010.10.050 21056648

[17] Rao M, Mandage Y, Thanki K, Bhise S. Dissolution improvement of simvastatin by surface solid dispersion technology. Diss Technol. 2010; 17: 27–34.

[18] Karolewicz B, Gajda M, Owczarek A, Pluta J, Gorniak A. Physicochemical and dissolution studies of simvastatin solid dispersions with Pluronic F127. Pharmazie. 2014; 69: 589–594. 25158569

[19] Craye G, Lobmann K, Grohganz H., Rades T., and Laitinen R., “Characterization of amorphous and co-amorphous simvastatin formulations prepared by spray drying”, *Molecules*, vol. 20, pp. 21532–21548, 2015. 10.3390/molecules201219784 26633346 PMC6332242

[20] Patil P, Patil V, Pradkar A. Formulation of a self-emulsifying system for oral delivery of simvastatin: in vitro and in vivo evaluation. Acta Pharm. 2007; 57: 111–122. 10.2478/v10007-007-0009-5 19839411

[21] Dixit RP, Nagarsenker MS. Optimized microemulsions and solid microemulsion systems of simvastatin: characterization and *in vivo* evaluation. J Pharm Sci. 2010; 99: 4892–4902. 10.1002/jps.22208 20648662

[22] Srinivas C, Sagar SV. Enhancing the oral bioavailability of simvastatin using microemulsion drug delivery system. Asian J Pharm Clin Res. 2012; 5: 134–139.

[23] Patil MS, Bavaskar KR, Girnar GA, Jain AS, Tekad AR. Preparation and optimization of simvastatin nanoparticle for solubility enhancement and *in vivo* study. Int J Pharm Res Dev. 2011; 2: 219–226.

[24] Akash C, Sudheer P. Lipid nanoparticulate system for simvastatin: a method for solubility enhancement. J Pharm Res. 2017; 11: 665–670.

[25] Jun SW, Kim M, Kim J, Park H, Lee S, Woo J, Hwang S. Preparation and characterization of simvastatin/hydroxypropyl-b-cyclodextrin inclusion complex using supercritical antisolvent (SAS) process. Eur J Pharm Biopharm. 2007; 66: 413–421. 10.1016/j.ejpb.2006.11.013 17240129

[26] Kulhari H, Poojaa D, Prajapatia SK, Chauhanb AS. Performance evaluation of PAMAM dendrimer based simvastatin formulations. Int J Pharm. 2011; 405: 203–209. 10.1016/j.ijpharm.2010.12.002 21145960

[27] Murtaza G. Solubility enhancement of simvastatin: a review. Acta Pol Pharm. 2012; 69: 581–590. 22876598

[28] Sun H, Gong J, Wang J. Solubility of lovastatin in acetone, methanol, ethanol, ethyl acetate, and butyl acetate between 283 K and 323 K. J Chem Eng Data. 2005; 50: 1389–1391.

[29] Sandeep K, Suresh P, Gupta GD. Effect of nonionic surfactant on the solubility and dissolution of simvastatin. Int Res J Pharm. 2011; 2: 100–102.

[30] Klevens HB. Solubilization. Chem Rev. 1950; 47: 1–74. 10.1021/cr60146a001 24538460

[31] Vinarov Z, Katev V, Radeva D, Tcholakova S, Denkov ND. Micellar solubilization of drugs: effect of surfactant and solubilizate molecular structure. Drug Dev Ind Pharm. 2018; 4: 677–686. 10.1080/03639045.2017.1408642 29164955

[32] Vinarov Z, Gancheva G, Burdzhiev N, Tcholakova S. Solubilization of itraconazole by surfactants and phospholipid-surfactant mixtures: interplay of amphiphile structure, pH and electrostatic interactions. J Drug Deliv Sci Technol. 2020; 57: E101688.

[33] International conference on harmonization (ICH), Q2 (R1): validation of analytical procedures–text and methodology, Geneva, Switzerland, 2005.

[34] Higuchi T, Connors KA. Phase-solubility techniques. Adv Anal Chem Inst. 1965; 4: 117–122.

[35] Shakeel F, Imran M, Abida, Haq N, Alanazi FK, Alsarra IA. Solubility and thermodynamic/solvation behavior of 6-phenyl-4,5-dihydropyridazin-3(2H)-one in different (Transcutol + water) mixtures. J Mol Liq. 2017; 230: 511–517.

[36] Shakeel F, Alshehri S, Ibrahim MA, Elzayat EM, Altamimi MA, Mohsin K, Alanazi FK, Alsarra IA. Solubility and thermodynamic parameters of apigenin in different neat solvents at different temperatures. J Mol Liq. 2017; 234: 73–80.

[37] Almarri F, Haq N, Alanazi FK, Mohsin K, Alsarra IA, Aleanizy FS, Shakeel F. Solubility and thermodynamic function of vitamin D3 in different mono solvents. J Mol Liq. 2017; 229: 477–481.

[38] Shakeel F, Haq N, Alanazi FK, Alsarra IA. Solubility and thermodynamics of apremilast in different mono solvents: determination. correlation and molecular interactions. Int J Pharm. 2017; 523: 410–417. 10.1016/j.ijpharm.2017.03.067 28359817

[39] Ahad A, Shakeel F, Alfaifi OA, Raish M, Ahmad A, Al-Jenoobi FI, Al-Mohizea AM. Solubility determination of raloxifene hydrochloride in ten pure solvents at various temperatures: thermodynamics-based analysis and solute-solvent interactions. Int J Pharm. vol. 544, pp. 165–171, 2018. 10.1016/j.ijpharm.2018.04.024 29679751

[40] Zhu QN, Wang Q, Hu YB, Abliz X. Practical determination of the solubility parameters of 1-alkyl-3-methylimidazolium bromide ([CnC1im]Br, n = 5, 6, 7, 8) ionic liquids by inverse gas chromatography and the Hansen solubility parameter. Molecules. 2019; 24: E1346. 10.3390/molecules24071346 30959775 PMC6479879

[41] Alanazi A, Alshehri S, Altamimi M, Shakeel F. Solubility determination and three dimensional Hansen solubility parameters of gefitinib in different organic solvents: experimental and computational approaches. J Mol Liq. 2020; 299: E112211.

[42] Shakeel F, Haq N, Alshehri S. Solubility data of bioactive compound piperine in (Trnascutol + water) mixtures: computational modeling, Hansen solubility parameters and mixing thermodynamic parameters. Molecules. 2020; 25: E2743. 10.3390/molecules25122743 32545724 PMC7355804

[43] Ruidiaz MA, Delgado DR, Martínez F, Marcus Y. Solubility and preferential solvation of indomethacin in 1,4-dioxane + water solvent mixtures. Fluid Phase Equilib. 2010; 299: 259–265.

[44] Hildebrand JH, Prausnitz JM, Scott RL. Regular and related solutions. Van Nostrand Reinhold, New York, 1970.

[45] Manrique YJ, Pacheco DP, Martínez F. Thermodynamics of mixing and solvation of ibuprofen and naproxen in propylene glycol + water cosolvent mixtures. J Sol Chem. 2008; 37: 165–181.

[46] Apelblat A, Manzurola E. Solubilities of o-acetylsalicylic, 4-aminosalicylic, 3,5-dinitrosalicylic and p-toluic acid and magnesium-DL-aspartate in water from T = (278–348) K. J Chem Thermodyn. 1999; 31: 85–91.

[47] Manzurola E, Apelblat A. Solubilities of L-glutamic acid, 3-nitrobenzoic acid, acetylsalicylic, p-toluic acid, calcium-L-lactate, calcium gluconate, magnesium-DL-aspartate, and magnesium-L-lactate in water. J Chem Thermodyn 2002; 34: 1127–1136.

[48] Alshehri S, Haq N, Shakeel F. Solubility, molecular interactions and mixing thermodynamic properties of piperine in various pure solvents at different temperatures. J Mol Liq. 2018; 250: 63–70.

[49] Holguín AR, Rodríguez GA, Cristancho DM, Delgado DR, Martínez F. Solution thermodynamics of indomethacin in propylene glycol + water mixtures. Fluid Phase Equilib. 2012; 314: 134–139.

[50] Krug RR, Hunter WG, Grieger RA. Enthalpy-entropy compensation. 2. Separation of the chemical from the statistic effect. J Phys Chem. 1976; 80: 2341–2351.

[51] Alshahrani SM, Shakeel F. Solubility data and computational modeling of baricitinib in various (DMSO + water) mixtures. Molecules 2020; 25: E2124. 10.3390/molecules25092124 32370021 PMC7249174

