# Supplementary material for: Solubilization and thermodynamic properties of simvastatin in various micellar solutions of different non-ionic surfactants: Computational modeling and solubilization capacity
Source: PLoS One. 2021 Apr 8;16(4):e0249485. doi: 10.1371/journal.pone.0249485 (PMC8031458; doi:10.1371/journal.pone.0249485)
Supplement: S2 Fig — (DOCX) [file pone.0249485.s002.docx]

**
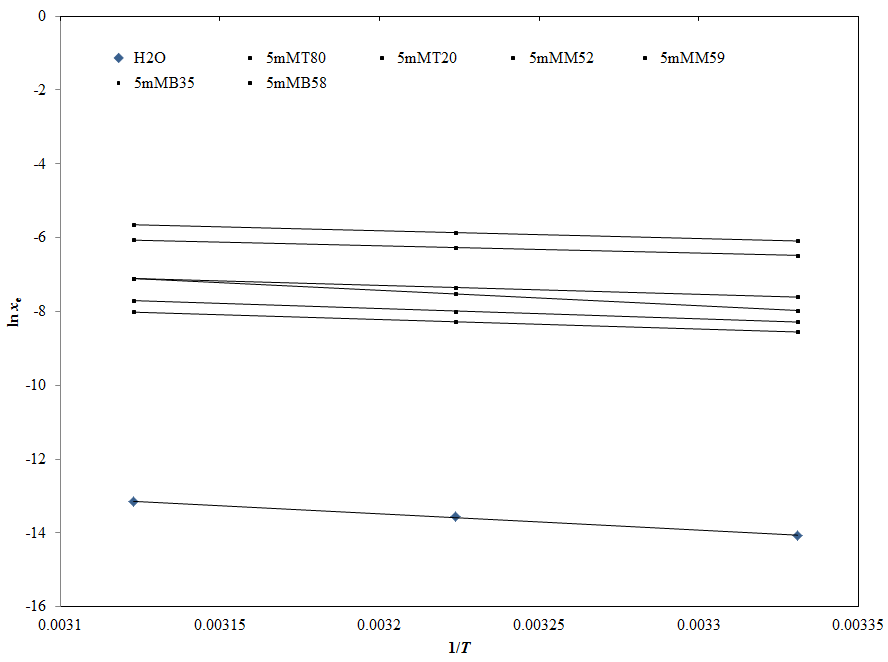
**

**S2 Fig.** Correlation of ln *x*_e_ values of SIM with van’t Hoff model in H_2_O and 5 mM molar solution of various non-ionic surfactants as a function of 1/*T*; symbols represent the experimental solubilities of SIM and solid lines represent the solubilities of SIM calculated by van’t Hoff model.
